# *Lactobacillus acidophilus* LA-5 Ameliorates Inflammation and Alveolar Bone Loss Promoted by *A. actinomycetemcomitans* and *S. gordonii* in Mice and Impacts Oral and Gut Microbiomes

**DOI:** 10.3390/microorganisms12040836

**Published:** 2024-04-22

**Authors:** Manuela R. Bueno, Fernando H. Martins, Catarina M. Rocha, Dione Kawamoto, Karin H. Ishikawa, Ellen S. Ando-Suguimoto, Aline R. Carlucci, Leticia S. Arroteia, Renato V. Casarin, Marcia P. A. Mayer

**Affiliations:** 1 Department of Microbiology, Institute of Biomedical Sciences, University of São Paulo, São Paulo 05508-000, SP, Brazil; fernando.martins@alumni.usp.br (F.H.M.); mpamayer@icb.usp.br (M.P.A.M.); 2 Department of Periodontology, Faculdade São Leopoldo Mandic, Campinas 13045-755, SP, Brazil; 3 Department of Stomatology, School of Dentistry, University of São Paulo, São Paulo 05508-220, SP, Brazilesa.2406@gmail.com (E.S.A.-S.); 4 Department of Prosthesis and Periodontology, School of Dentistry, University of Campinas, Campinas 13083-875, SP, Brazil; leticia.sandoli@hotmail.com (L.S.A.);

**Keywords:** *Aggregatibacter actinomycetemcomitans*, immune modulation, lactobacilli, periodontitis, probiotics

## Abstract

The benefits of probiotics on dysbiotic microbiomes and inflammation are dependent on the tested strain, host factors, and the resident microbiome. There is limited knowledge on the effects of probiotics in *A. actinomycetemcomitans*-associated periodontitis. Thus, *Lactobacillus acidophilus* LA5 (LA5) was orally inoculated for 30 days in C57Bl/6 mice infected with *A. actinomycetemcomitans* JP2 (Aa) and *S. gordonii* (Sg). Alveolar bone loss, gingival gene expression, and oral and gut microbiomes were determined. LA5 controlled bone loss in Aa+Sg-infected mice, downregulated the expression of *Il-1β* and upregulated *Il-10* in gingival tissues, and altered the oral and gut microbiomes. LA5 increased the diversity of the oral microbiome of Aa+Sg infected mice, and Aa+Sg and Aa+Sg+LA5 oral or gut microbiomes clustered apart. LA5 induced shifts in Aa+Sg infected mice by increasing the abundance of *Muribaculaceae* and decreasing *Bifidobacteriaceae* in the oral cavity and increasing the abundance of *Verrucomicrobiae* and *Eggerthellales* in the gut. In conclusion, LA5 oral administration controls experimental Aa-associated periodontitis by altering inflammatory gene expression and the oral and gut microbiomes.

## 1. Introduction

Periodontal diseases (PD) are inflammatory conditions of the tooth-supporting structures induced by dysbiotic subgingival biofilms. The Gram-negative facultative bacterium *Aggregatibacter actinomycetemcomitans* (Aa) is involved in the polymicrobial community of periodontitis, especially of rapidly progressing disease with molar-incisor pattern (MIP) affecting young subjects (previously known as localized aggressive periodontitis) [1,2]. *A. actinomycetemcomitans* is 50-times more abundant in MIP sites than in subgingival sites of age-/race-matched healthy controls, and dysbiosis is seem not only in the oral but also in the gut microbiome of these diseased patients [2]. *A. actinomycetemcomitans*-associated periodontitis usually requires systemic antibiotic for successful treatment [3] and life-long supportive periodontal therapy [4].

Animal and clinical studies indicated that interventions with beneficial bacteria such as probiotics would be able to control the dysbiotic biofilm and modulate host response in periodontitis [5]. Recent meta-analyses data revealed that the oral administration of probiotics results in an improvement in clinical parameters and immunological biomarkers in gingivitis patients and periodontally healthy subjects [6]. These data also showed that the administration of probiotics as adjuvant treatment in combination with scaling and root planning can improve clinical parameters, reduce proinflammatory markers, and change the microbial profile in chronic periodontitis patients [6,7]. However, these randomized clinical trials of probiotics may not be comparable, since variables such as criteria for patient selection, probiotic strain, dose, frequency, and period of probiotic treatment can potentially affect the experimental outcomes.

The benefits of probiotics for each disease are specific to each isolate and differ even between isolates of the same species [8]. Since probiotic health benefits should be predictable based on the strain’s properties and their underlying mechanisms [9], we selected the commercially available probiotic strain *Lactobacillus acidophilus* LA5 for this study due to its antimicrobial and immunomodulatory properties. The data of in vitro analyses revealed that *L. acidophilus* LA5 can impair the establishment of pathogens in a subgingival multispecies biofilm model [10], reduce the apoptosis of infected gingival epithelial cells [11], regulate the transcription of bacteria virulence factors [12,13], and reduce the dysbiosis of the diabetic gut microbiota [14]. *L. acidophilus* LA5 modulatory properties comprise the attenuation of epithelial cells response to *Porphyromonas gingivalis* [13] and to *A. actinomycetemcomitans* [11] and of dendritic cells to LPS [15], whereas this strain induced a high response of otherwise non stimulated CD14 + monocytes [16]. We have also recently shown that LA5 was able to control bone destruction induced by a pathogenic consortium formed by *P. gingivalis, Prevotella intermedia, Fusobacterium nucleatum*, and *S. gordonii* in a murine periodontitis model [17]. Thus, to evaluate whether *L. acidophilus* LA5 would also impact periodontitis associated with *A. actinomycetemcomitans*, such as MIP periodontitis, we tested its effect in an *A. actinomycetemcomitans* and *S. gordonii* periodontitis experimental model by evaluating the alveolar bone loss, expression of inflammatory mediators and pathogens’ recognition patterns in the gingiva, as well as the oral and gut microbiomes.

## 2. Materials and Methods

### 2.1. Animals and Group Allocation

Thirty-two 4-week-old old C57Bl/6 male mice, bred under specific pathogen-free conditions, were acquired from the Central Facility of School of Medicine, USP, and kept in the mouse breeding facility of the Department of Microbiology and Parasitology, Institute of Biomedical Sciences, USP. Animals were kept in microisolators, with an artificial light–dark cycle of 12 h and room temperature of 22 °C, with water and food available ad libidum and randomly allocated in four groups (n = 8): non-infected negative control (SHAM), positive control (Aa+Sg), probiotic control (LA5) and test group (Aa+Sg+LA5). The animals were monitored for weight gain, loss of mobility, and skin appearance throughout the experimental period. Procedures were performed following National Institutes of Health Guidelines for Experimental Animal Welfare and approved by the Institutional Animal Care and Use Committee (ICB/USP numbers: 3104200220 and 4828281020).

### 2.2. Blinding

Each animal was assigned a temporary random number within the group. Based on their position on the rack, cages were given a numerical designation. For each group, a cage was selected randomly from the pool of all cages. Blinding was carried out during the allocation, evaluation of the results, and data analysis. Blindness was unfeasible during the experiment since the bacterial suspensions differed in color from the vehicle.

### 2.3. Exclusion Criteria

Animals presenting alteration in growth, weight and/or physical defects at baseline were excluded.

### 2.4. Sample Size

Alveolar bone loss was the primary outcome and therefore used for sample size calculation. A pilot study was conducted taking into consideration a difference in the bone volume of 4719 cubic pixels at a standard area, and a sample size of 7.84 animals was adequate to obtain a Type I error rate of 5% and power greater than 80% [18,19]. Thus, each group was formed by 8 animals.

### 2.5. Bacteria Strains and Culture Conditions

*Lactobacillus acidophilus* LA-5™ (CHR Hansen Holding A/S, Hørsholm, Denmark) was used as the probiotic strain. The microbial consortium consisted of *A. actinomycetemcomitans* strain JP2 [20] and *S. gordonii* DL1 [21].

LA5 was cultured in MRS Lactobacilli agar and broth, *A. actinomycetemcomitans* in tryptone soy agar with 0.5% yeast extract or brain heart infusion (BHI) broth, and *S. gordonii* in BHI agar or broth, incubated at 37 °C, 5% CO_2_.

Standard broth cultures were obtained, and cells were harvested and resuspended in 500 μL lyophilization solution (10% skin milk with 5% L-Glutamic acid monosodium salt hydrate, and 5% dithiothreitol) (Sigma-Aldrich, Darmstadt, Germany). Aliquots were lyophilized (Freezone Triad Freezer Dryers, Labconco, Kansas City, MI, USA) and maintained at −80 °C. Lyophilized bacteria of the microbial consortium were inoculated in BHI broth, incubated for 6 h under 37 °C/5% CO_2_ to recover to physiological state prior being inoculated. Viability was estimated for each lot.

### 2.6. Experimental Treatments

Groups Aa+Sg and Aa+Sg+LA5 received 50 µL aliquots containing 1 × 10^9^ CFU *A. actinomycetemcomitans* and 1 × 10^8^ CFU *S. gordonii* in PBS/1.5% carboxymethylcellulose, into the oral cavity with the aid of a gavage needle, three times a week for four weeks [22]. These groups also received, under anesthesia, a palatal injection of 10 µL containing 1 × 10^7^ CFU *A. actinomycetemcomitans* in PBS in the interproximal gingiva between the first and second molars on the left hemimaxilla [23] at days 01, 03, and 05 of the experimental period. The non-infected group (SHAM) and the probiotic control group (LA5) were orally inoculated with PBS/1.5% carboxymethylcellulose and received a palatal injection with PBS at the same days and volumes used in the Aa+Sg infected groups.

**Figure 1:**
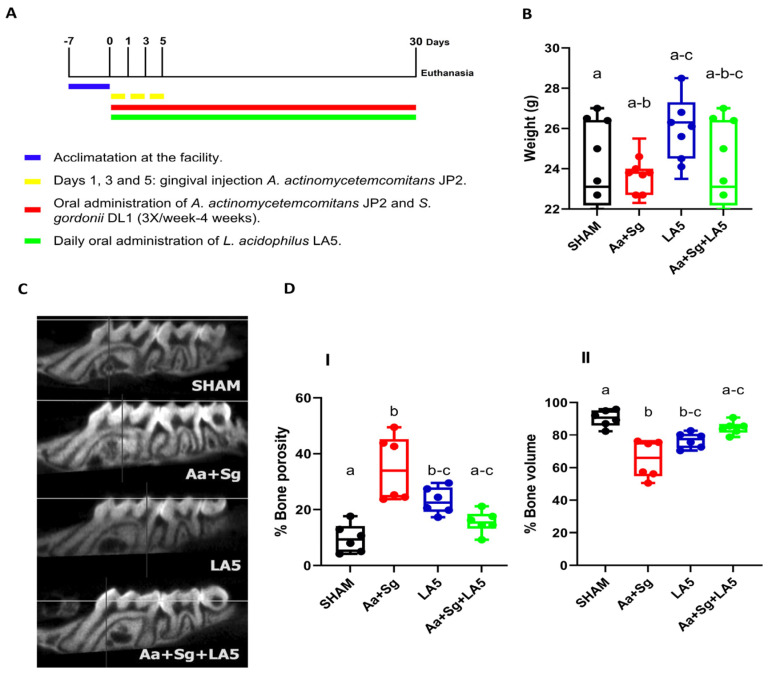
C57Bl/6 mice were allocated into four groups (n = 8) and submitted to different treatments for 30 days: negative control (SHAM); positive control: *A. actinomycetemcomitans* and *S. gordonii* (Aa+Sg); probiotic control: *L. acidophilus* (LA5), and test group: *A. actinomycetemcomitans*, *S. gordonii* and *L. acidophilus* LA5 (Aa+Sg+LA5). (**A**) Study design. Control groups received the vehicles. (**B**) Animals weight in grams at the end of the experimental period. (**C**) Alveolar bone analysis determined by microtomography in the interproximal region of first and second molar at the left maxilla. Representative image of the alveolar bone of different groups. (**D**) Percentage of alveolar bone porosity (**I**) and percentage of alveolar bone volume (**II**). Different letters indicate statistical difference among groups. Kruskal–Wallis, post-hoc Dunn (*p* < 0.05).

Groups LA5 and Aa+Sg+LA5 received the probiotic daily in the oral cavity for 30 days, in 50 µL aliquots containing 1 × 10^8^ CFU of LA5 in PBS/1.5% carboxymethylcellulose. SHAM and Aa+Sg groups received the vehicle (Figure 1A).

### 2.7. Euthanasia and Samples Collection

After 30 days, the animals were anesthetized (ketamine (100 mg/kg IP) + xylazine (10 mg/kg IP)) and sacrificed by exsanguination.

Oral biofilm was obtained with a microbrush, the content of the jejunum was obtained with a spatula and both samples were kept in TRIS-EDTA, pH 7.4, for microbiome analysis. The gingival tissue around the buccal and palatal surfaces of the left molars was removed and stored in RNAlaterTM Stabilization Solution (Invitrogen Life Technologies, Carlsbad, CA, USA) for gene expression analyses. The left hemimaxilla was kept in 4% formaldehyde solution for 24 h, transferred to PBS, and stored for alveolar bone analysis.

### 2.8. Alveolar Bone Loss Analysis

Alveolar bone resorption was determined by micro tomography (SkyScan 1174 version 1.1, Kontich, Belgium) at 45 kV voltage, 550 uA current, 8.71 μm pixel size, 0.2 mm aluminum filter. The left hemimaxillae were scanned, and a blinded examiner selected a standard area of 60 × 30 pixel (Roi) at the interproximal region between the first and second molar from the second molar cementoenamel junction in 15 coronal sections. The images were analyzed by calculating percentages of bone volume and porosity using CT Analyzer software Version 1.15.4.0, SkyScan.

### 2.9. Gene Expression in Gingiva

RNA was extracted using Trizol LS Reagent (Invitrogen Life Technologies, Carlsbad, CA, USA) in a cell disrupter (BioSpec 3110BX Mini-BeadBeater-1 High Energy Cell Disrupter, Campinas, SP, Brazil) for 20 s, twice. After deoxyribonuclease (Ambion™ DNase I, Invitrogen Life Technologies) treatment, cDNA was obtained using the SuperScriptTM ViloTM Synthesis Kit for RT-PCR (Invitrogen Life Technologies). Quantitative PCR was performed in StepOne Plus System thermocycler (Applied Biosystems, Foster City, CA, USA) with 100 ng cDNA using TaqMan™ Gene Expression Assay (Invitrogen by Thermo Fisher Scientific, Vilnius, Lithuania). Commercial Taqman primers and probes (Invitrogen Life Technologies, Carlsbad, CA, USA) comprised *Tlr-2* (Mm01213946_g1), *Tlr-4* (Mm00445273_m1), *Il-1β* (Mm00434228_m1), *Il-10* (Mm01288386_m1), *Tnf* (Mm00443258_m1), *β-actin* (Mm00607939_s1), and *Gapdh* (Mm99999915_g1). Relative expression of target genes was calculated by the ΔΔCT method, using *β-actin* and *Gapdh* as endogenous controls [24], and expressed as fold changes in relation to control group (SHAM).

### 2.10. Oral and Gut Microbiomes

DNA from oral biofilm and gut samples of six animals per group was extracted using the Master pureTM DNA Purification Kit (Epicentre^®^ Illumina Company, Madison, WI, USA). A barcoded primer set Bakt_341F CCTACGGGNGGCWGCAG and Bakt_805R GACTACHVGGGTATCTAATCC [25] was used to amplify the hypervariable V3–V4 region of 16SrRNA. DNA was sequenced by ByMyCell (Ribeirão Preto, São Paulo, Brazil) using the Illumina MiSeq 2 × 250 platform. Data were submitted to Sequence Read Archive (SRA) under BioProject identification #PRJNA994574.

### 2.11. Statistical Analysis

Data normality was checked using the Kolmogorov–Smirnov statistical test with Lilliefors correlation, and homogeneity of variances was assessed using the F test. Parametric data were analyzed by Kruskal–Wallis, followed by Dunn’s post-hoc test. Statistical significance was set at *p* < 0.05. The GraphPad Prisma^®^ Version 9.0.0 program was used (GraphPad Software, La Jolla, CA, USA).

Microbiome data were analyzed using Qiime 2 2022.8 [26]. Demultiplexed sequences and reads were filtered using Dada 2, and quality score threshold = 25. Trimmed sequences were clustered into amplicon sequence variants (ASVs), and taxonomy was assigned using Silva138 database [27,28]. Alpha diversity indices (Faith, Pielou and Shannon), and Beta diversity by Weighted and Unweighted UniFrac distances were calculated. Clustering was visualized by principal Coordinates Analysis (PCA) [26,29] and differences among groups determined by Permanova (999 permutations). Differences in mean relative abundance of taxa were determined by ANCOM (analysis of composition of microbiomes) [30], using 75% as the empirical cut-off value. The expanded HOMD (eHOMD) database was assessed to search for the inoculated species.

## 3. Results

### 3.1. Animals Changes

Animals did not exhibit any changes in fur, skin, and mobility throughout the experiment. There were no differences in animals’ weight at baseline. There was a trend to increased final weight in the groups that received LA5 (LA5 and Aa+Sg+LA5) when compared to SHAM and Aa+Sg, respectively (Figure 1B).

### 3.2. Alveolar Bone Loss

The inoculation of Aa+Sg induced alveolar bone loss. Administration of LA5 in otherwise non-infected animals (probiotic control group-LA5) also showed some degree of destruction compared to SHAM. However, administration of LA5 to Aa+Sg-infected animals (Aa+Sg+LA5) prevented bone destruction induced by Aa+Sg infection (Figure 1C,D).

### 3.3. Transcription Analysis in Gingival Tissue

**Figure 2:**
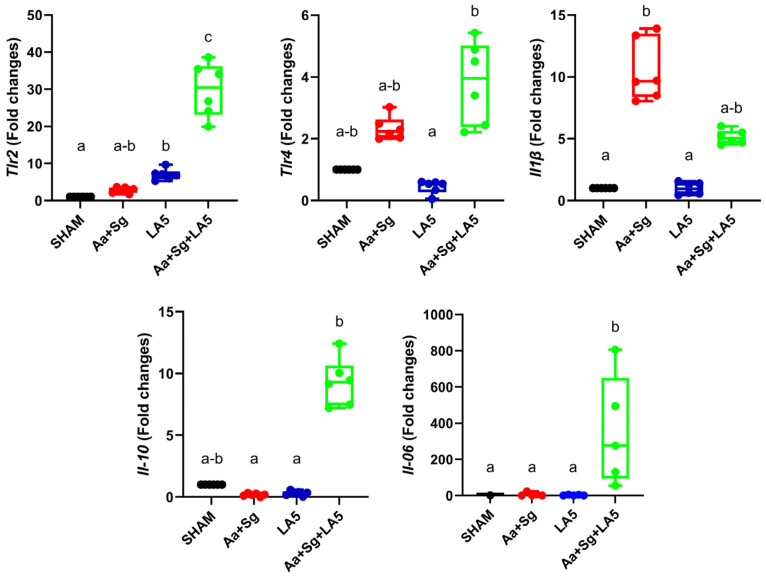
Relative transcription of genes encoding receptors *Tlr-2* and *Tlr-4* and cytokines *Il-1β*, *Il-10*, and *Il-6* in gingival tissue of C57Bl/6 mice submitted to different treatments: Groups (n = 6): negative control (SHAM); positive control: *A. actinomycetemcomitans* and *S. gordonii* (Aa+Sg); probiotic control: *L. acidophilus* (LA5), and test group: *A. actinomycetemcomitans*, *S. gordonii* and *L. acidophilus* LA5 (Aa+Sg+LA5). Data on target genes were normalized to mRNA levels of Gapdh and/or β-actin reference genes (internal controls). Different letters indicate statistical difference among groups. Kruskal–Wallis, post-hoc Dunn (*p* < 0.05).

Infection with Aa+Sg increased mRNA levels of *Il-1β*. Administration of LA5 to otherwise non-infected animals led to the upregulation of *Tlr2* but did not alter the transcription of other studied genes. There was no difference in *Tnf* expression levels among groups. However, administration of LA5 to Aa+Sg-infected animals led to the upregulation of *Tlr2* and *Tlr4*, downregulation of Il-1β, and upregulation of *Il-10* and *Il-6* (Figure 2).

### 3.4. Oral and Gut Microbiomes

Oral biofilm and gut samples from six animals/group were evaluated for the microbiome analyses. However, the data were obtained only from four animals of the SHAM group due to the poor recovery of DNA from oral biofilm samples. The total number of sequences obtained after the amplification of 16SrRNA of oral and gut samples was 7893.351 (max 386,428 and min 92,229). After filtering and removal of chimeras, the total number was 257,7399 for oral (max 147,037 and min 82,876) and 1,609,759 for gut samples (max 229,627 and min 4859).

**Figure 3:**
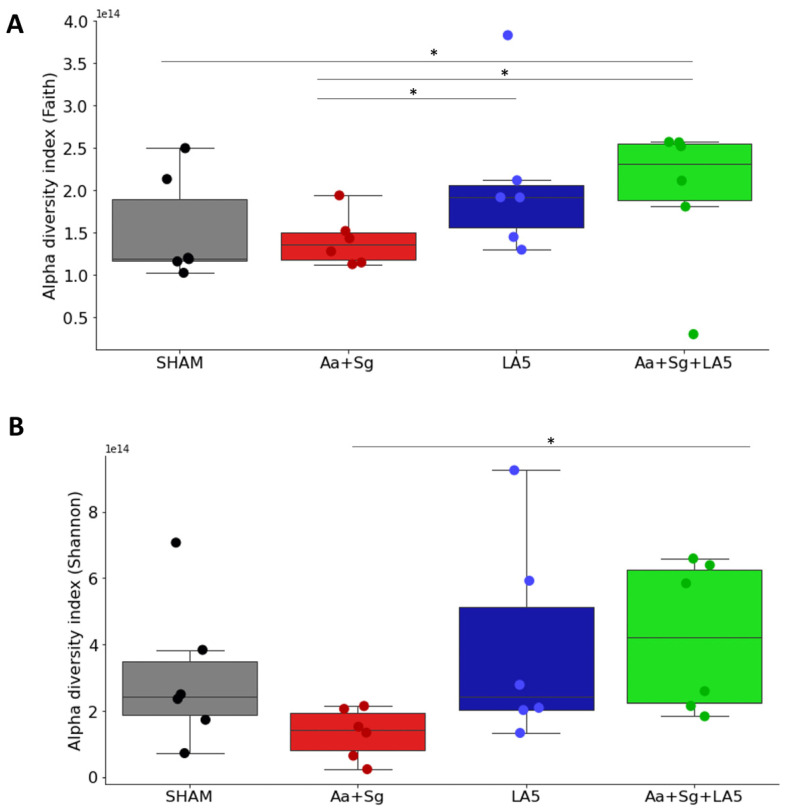
Alpha diversity analyses of the oral biofilm microbiome of C57Bl/6 mice of the following groups (n = 6): negative control (SHAM); positive control: *A. actinomycetemcomitans* and *S. gordonii* (Aa+Sg); probiotic control: *L. acidophilus* (LA5), and test: *A. actinomycetemcomitans*, *S. gordonii* and *L. acidophilus* LA5 (Aa+Sg+LA5). (**A**) Richness (Faith’s PD) index and (**B**) Richness Shannon index analyses * Kruskal–Wallis (*p* ≤ 0.05).

Inoculation of Aa+Sg tended to decrease alpha diversity indices (not significant) of oral biofilms. However, administration of LA5 induced a more diverse phylogenetic community (Faith) and increased the richness (Shannon) of the oral microbiome of Aa+Sg-infected mice (Figure 3A,B). There were no differences in Alpha diversity indices (Faith, Pielou, and Shannon) of the gut microbiome among groups.

**Figure 4:**
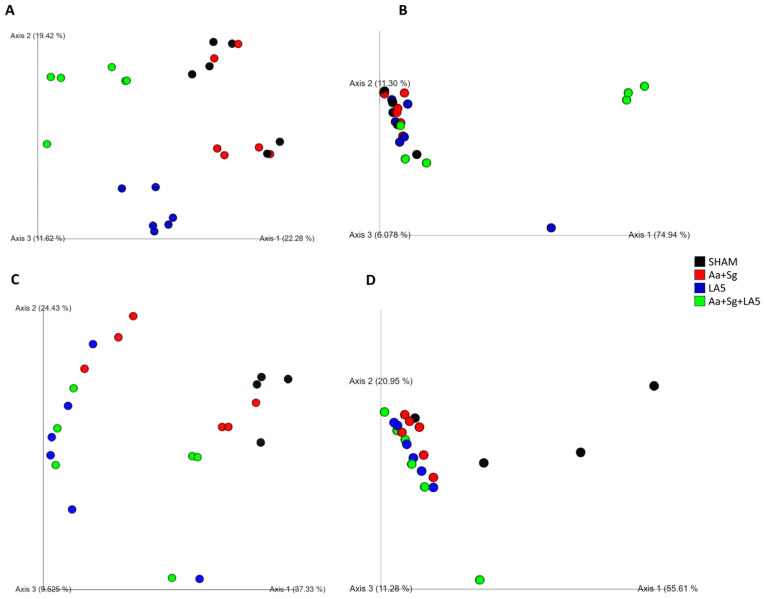
Treatment with LA5 induced alterations in the oral and gut microbiome of infected animals. Principal coordinate analysis (PCoA), based on unweighted (**A**) and weighted (**B**) UniFrac distance metrics performed on oral biofilm microbial communities and unweighted (**C**) and weighted (**D**) UniFrac distance metrics on gut microbial communities of C57Bl/6 mice from different groups (n = 6): negative control (SHAM), positive control (*A. actinomycetemcomitans* and *S. gordonii*-Aa+Sg), probiotic control (*L. acidophilus*-LA5), and test group (Aa+Sg+LA5). The PCoA revealed significant changes in the oral and gut composition of C57Bl/6 mice. Treatments LA5 and Aa+Sg+LA5 exhibited similar effects, with Aa+Sg+LA5 causing the most significant shift. These findings underscore treatment-specific impacts on oral microbial communities (PERMANOVA, 999 permutations, *p* < 0.01).

**Figure 5:**
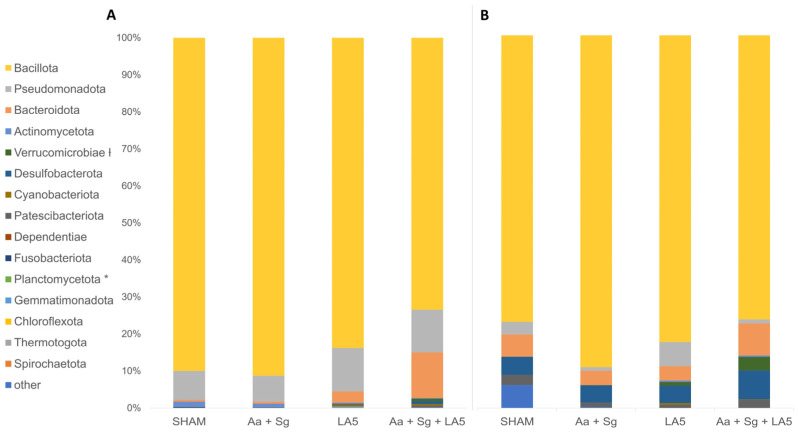
Microbial abundance of bacteria at the phylum level in the oral (**A**) and gut (**B**) microbiomes of C57Bl/6 mice. The following groups were examined: negative control (SHAM), positive control (*A. actinomycetemcomitans* and *S. gordonii*-Aa+Sg), probiotic control (*L. acidophilus*-LA5), and test group (Aa+Sg+LA5). ANCOM (*) indicates statistical differences in the oral microbiome for Planctomycetota. (ƚ) denotes statistical differences in the gut microbiome for Verrucomicrobiota. The Kruskal–Wallis test was performed, with utilized *p*-values: *p* < 0.05, *p* < 0.01, and *p* < 0.001.

Inoculation of the microbial consortium Aa+Sg did not alter the population structure of the oral microbiome, since the SHAM and Aa+Sg microbial communities did not differ based on Unweighted and Weighted Unifrac distances (Figure 4). However, infection with Aa+Sg led to a shift in the gut microbiome compared to SHAM-infected animals based on Unweighted and Weighted Unifrac distances measurements (Figure 5).

Inoculation of LA5 either alone (probiotic control group) or in mice infected with Aa+Sg altered the population structure of the oral microbiome, based on the Unweighted and Weighted Unifrac distances, as shown in the PCoA plots (Figure 4A,B).

The probiotic LA5 also interfered with the structure of the gut microbiome. Beta diversity analysis based on Unweighted Unifrac distances demonstrated that all groups clustered apart, except for groups LA5 and Aa+Sg+LA5 (Figure 4C). Analyses based on Weighted Unifrac distances revealed that the microbial communities in the gut of groups SHAM, Aa+Sg and Aa+Sg+LA5 differed from each other, and LA5 differed from SHAM, as visualized in PCoA plots and determined by PERMANOVA (Figure 4D).

**Figure 6:**
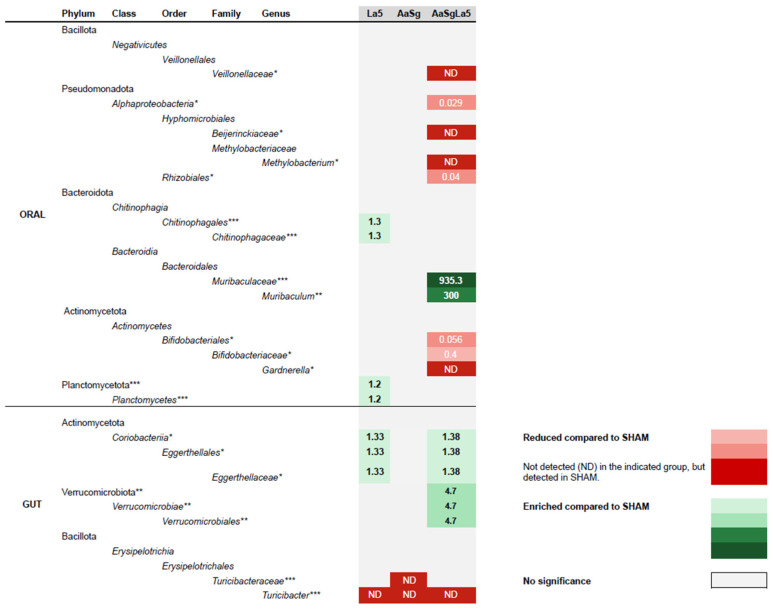
Disparities in the relative abundance (expressed as fold changes relative to SHAM) of bacterial taxa in the oral and gut microbiomes of C57Bl-6 mice across distinct groups: negative control (SHAM), positive control (*A. actinomycetemcomitans* and *S. gordonii*-Aa+Sg), probiotic control (*L. acidophilus*-LA5), and test group (Aa+Sg+LA5). Data were subjected to analysis via ANCOM, with the 75th percentile of the W distribution serving as the empirical cut-off value. Statistically significant differences among groups were validated using Kruskal–Wallis (* *p* < 0.05, ** *p* < 0.01, *** *p* < 0.001).

Bacillota was the most abundant phylum in all oral and gut communities independently on the treatment (Figure 5A,B). ANCOM revealed differences in RA of Planctomycetota among oral samples (Figure 5A) and Verrucomicrobiae in gut samples (Figure 5B). Furthermore, differences in RA of several taxa in oral and gut samples at lower taxonomic levels were demonstrated (Figure 6).

The eHOMD database analyses indicated that Aa was detected at a low abundance (~0.02%) in three out of six oral samples of the Aa+Sg group but in no other oral or gut samples. Nevertheless, *S. gordonii* and *L. acidophilus* were not detected in any of the studied oral and gut samples.

## 4. Discussion

We have tested the effect of the probiotic strain *L. acidophilus* LA5 in an experimental model of periodontitis induced by the pathogen *A. actinomycetemcomitans* associated with *S. gordonii* due to their synergistic effect [31,32]. Although most species of oral streptococci of sanguinis-mitis groups, which includes *S. gordonii*, are commensals, *S. gordonii* can be considered an “accessory pathogen” [31]. Cooperation between *S. gordonii* and *A. actinomycetemcomitans* increased the survival and persistence of *A. actinomycetemcomitans* and promoted its virulence in a murine abscess model [33]. This cooperation involves the production of L-lactate and H_2_O_2_ by *S. gordonii*, providing nutrients for *A. actinomycetemcomitans* [34] but favoring its dispersal throughout the oral cavity [35].

*Aggregatibacter actinomycetemcomitans* was detected in the oral biofilm of three out of six mice of the Aa+Sg-infected group, suggesting successful colonization in accordance with data of in vitro and in vivo abscess experimental model [32,33]. Furthermore, Aa+Sg-infected mice exhibited alveolar bone loss and the upregulation of Il-1β in gingival tissues. Moreover, oral administration of *L. acidophilus* LA5 was able to control alveolar bone loss induced by Aa+Sg infection, reduced Aa to undetectable levels in the oral biofilm of all studied animals, and altered the transcriptional profile of gingival tissues.

Administration of LA5 to otherwise non-infected animals resulted in a slight increase in *Tlr2* mRNA levels, but no other evident result was observed in the mice of the LA5 group, except for a shift in the oral and gut microbiomes. Thus, the increase in Tlr2 transcription promoted by LA5 in the gingival tissues of mice may increase surveillance in the oral mucosa, maintaining a healthy associated microbiota in balance with the host.

On the other hand, the administration of LA5 to Aa+Sg-infected animals led to the upregulation of *Tlr2* and *Tlr4*, suggesting further increased surveillance in the oral mucosa, but was able to reduce inflammation, as observed by the altered transcription profile of inflammatory mediators. The capacity to differentially modulate Toll-like receptors (TLRs) is considered an important characteristic of immunobiotic strains [36]. Although the mechanisms underlying the modulation of inflammation induced by *L. acidophilus* LA5 are not fully understood, our previous data in LPS-mature dendritic cells indicate that *L. acidophilus* LA5 is able to alter the transcription of several genes involved in TLRs signaling and in the regulation of NF-kappa B activation [15]. *Tlr2* upregulation is a common trait observed after probiotics treatment in the gut [37], and LA5 was shown to induce *Tlr2* expression in gingival epithelial cells [11,13]. This mechanism is relevant to the anti-inflammatory effects of lactobacilli probiotics, leading to an increased recognition of cell surface lipoproteins and teichoic acid, as well as the production of negative regulators of the NF-κB signaling pathway in a TLR2-dependent manner [38,39]. In contrast, the upregulation of *Tlr4* promoted by the probiotic in Aa+Sg-infected mice was unexpected since *L. acidophilus* LA5 decreased *Tlr4* expression in gingival epithelial cells and monocytes in vitro [11,40], and the present data indicate that the probiotic altered cytokines transcription profile toward an anti-inflammatory effect.

The downregulation of *Il-1β* and upregulation of *Il-10* promoted by LA5 in Aa+Sg infected mice are attractive effects to modulate bone resorption and are expected in successful periodontal treatment in humans [41,42,43]. On the other hand, the effect of the increased *Il-6* transcription levels induced by the probiotic is less clear. Il-6 is a proinflammatory cytokine, and treatment with monoclonal antibodies against Il-6 receptor resulted in decreased periodontal inflammation and improved periodontal status [43]. However, IL-6 increased response is a common feature after probiotic treatment and is associated with an increased production of IgAS against pathogens in Peyer Patches in the gut [44] in a TLR2-dependent mechanism [45]. The overall attenuation of inflammation and reduced bone resorption promoted by LA5 in vivo after Aa+Sg challenge could not be predicted by in vitro studies since LA5 induced *Il-1β* and *Il-6* transcription and Il-1β release by DCs and monocytes [16,40] but reduced their expression by epithelial cells [11,13].

Infection with Aa+Sg promoted changes in the oral microbiome. However, administration of *L. acidophilus* LA5 induced more shifts in the oral microbiome than infection with the pathobionts. LA5 administration increased diversity of Aa+Sg-infected mice and impacted their oral microbiomes, although there were no shifts in the abundance of the main phyla Bacillota (former Firmicutes), Pseudomonadota (former Proteobacteria), or Bacteroidota (former Bacteroidetes) [46]. Dysbiosis of the oral microbiome is characterized by increased abundance of opportunistic/inflammophilic organisms and decreased abundance of commensals species. However, the administration of LA5 to Aa+Sg-infected animals resulted in an decreased abundance of some Alpha Pseudomonadota, *Veillonellaceae*, and *Bifidobacteriaceae* with no known pathogenic potential in the oral cavity, as well as an increased abundance of *Muribaculaceae*. *Muribaculaceae* is considered beneficial to the gut due to production of short-chain fatty acids (SCFA), especially propionate [47], and its abundance was increased by other probiotic lactobacilli [48]. The abundance of *Muribaculaceae* is reduced in inflammatory diseases [49,50,51], including in periodontitis [52], but its role in the oral cavity remains unknown.

Animal experimental studies have suggested that swallowing of oral bacteria results in dysbiosis in the gut and systemic inflammation [53]. These data are reinforced by human studies indicating an altered microbiome in the gut of periodontitis patients [2,54,55]. Our data indicated that infection with Aa+Sg altered the gut microbiome by increasing the abundance of *Eggerthelaceae* and decreasing the abundance of *Turicibacteraceae*.

The oral administration of LA5 was also able to alter the gut microbiome. Although LA5 viability may decrease throughout the passage in the hazardous environment of the stomach, its beneficial effects of LA5 toward a healthier gut microbiome were indicated by simulating the digestive system in vitro [14]. Our data indicated that the administration of LA5 increased the abundance of *Verrucomicrobiae* and *Eggerthelaceae* in the gut, especially of Aa+Sg-infected mice. The role of *Eggerthelaceae* in the gut is not fully understood, but its high abundance was associated to the beneficial effects of curcumin in a mouse model [50]. More importantly, members of the phylum Verrucomicrobiae improve the integrity of the intestinal barrier and regulate host metabolism and immunity [56]. Thus, our data corroborate the existing evidence that LA5 administration can enrich the gut microbiome with beneficial organisms related to health [44,45,57,58].

Overall, the probiotic treatment showed antimicrobial as well as anti-inflammatory effects, leading to the control of periodontitis induced by *A. actinomycetemcomitans*, as previously expected from in vitro studies [11,12]. However, these data should be interpreted under the limitations of the animal model. *A. actinomycetemcomitans*’ main virulence factor, the leukotoxin, does not affect murine cells [59], whereas adhesion to epithelial cells mediated by *A. actinomycetemcomitans*’ adhesins Aae and OMP100 is also impaired in mice [58,60]. Furthermore, interaction of *A. actinomycetemcomitans* with *S. gordonii* can alter the expression of key virulence factors of *A. actinomycetemcomitans* [33]. Another drawback from this model was observed when the administration of the probiotic alone also induced a slight alveolar bone resorption (Figure 1D), contrasting with our previous data, where LA5 induced no significant alveolar bole destruction in mice with a reduced microbiome [17].

Other uses of *L. acidophilus* LA5 include the control of diabetes [14], gestational diabetes [61], and *Clostridium difficile* infection [62]. Regarding periodontitis, this strain could control alveolar bone loss induced by experimental inoculation of *P. gingivalis* and other anaerobic organisms, as well as by *A. actinomycetemcomitans*, suggesting its beneficial effect against different forms of periodontitis in humans.

The ideal probiotic treatment for any infectious-inflammatory disease should be based not only on the nature of the disease but also on the initial resident microbiome prior to probiotic treatment, and other factors, such as genetic susceptibility and disease progression, should be considered. Future research should not only evaluate the efficacy of different probiotic regimens in randomized clinical trials but should also provide the principles to select the better probiotic regimen on an individual basis, focusing on the mechanisms underlying probiotics effects and on the factors involved in the success and failures of probiotic administration for each disease. Moreover, side effects should be observed as probably not all patients can be safely treated due to their systemic health [63].

## 5. Conclusions

Under the limitations of previous in vitro studies and of this in vivo experimental murine model, we can conclude that *L acidophilus* LA5 is a potential candidate to control periodontitis in humans due to its immunomodulatory properties, its antimicrobial effects against bacteria implicated in the disease, and its ability to alter the oral and gut microbiomes in experimental periodontitis. These results should be taken with reservation as future clinical trials should be performed to assess the therapeutic effects of *L. acidophilus* LA5 in the control of periodontal disease progression.

## Data Availability

Data are contained within the article.
